# US county-level prevalence and spatial distribution of optimal birth outcomes 2018–2019

**DOI:** 10.1038/s41598-022-20517-9

**Published:** 2022-10-03

**Authors:** Lauren Dyer, Caryn Bell, Susan Perez, Joia Crear-Perry, Katherine Theall, Maeve Wallace

**Affiliations:** 1 Mary Amelia Women’s Center, Department of Social, Behavioral and Population Sciences, Tulane University School of Public Health and Tropical Medicine, 1440 Canal St., New Orleans, LA 70112 USA; 2 National Birth Equity Collaborative, 4747 Earhart Blvd, New Orleans, LA USA; 3 Department of Public Health, California State University, Sacramento, 6000 J Street, Sacramento, CA 95819 USA

**Keywords:** Public health, Epidemiology

## Abstract

A shift in focus towards healthy reproductive outcomes may reveal opportunities for novel interventions and strategies to promote optimal health. Using variables from the National Center for Health Statistics restricted use natality files, we calculated Empirical Bayes smoothed (EBS) rates of optimal birth for the all live births—both overall and by maternal race/ethnicity—by applying the smoothing tool in GeoDa version 1.18.0.10 We defined counties achieving greater racial birth equity as those where the overall EBS optimal birth rate was greater than the national 75th percentile and the absolute difference between maternal racial/ethnic categories was smaller than the national 25th percentile difference. During the study period, 49.80% of overall births could be classified as an optimal birth according to the study definition. Of the 3140 US counties, only 282 (8.98%) appeared to advance White-Black equity in optimal births, and 205 (6.53%) appeared to advance White-Hispanic equity in optimal births. In the effort improve maternal health, we should focus not only on the absence of negative outcomes, but also the occurrence of positive outcomes. Our analytic results suggest that optimal births can be measured and that geographic inequities by race occur.

## Introduction

Similar to most public health studies, maternal and infant health research tends to use deficit models or to center on adverse outcomes. Work of this nature is invaluable given that adverse outcomes may represent life-course health experiences and can also serve as early indicators for other phenomena, such as chronic health conditions and even socioeconomic disparities. For instance, preterm birth and low birthweight have the potential to increase an infant’s risk of developing neurological disorders^1^. Likewise, maternal mortality has been recognized as a key indicator of population health and of overall social and economic development^2^. However, adverse outcomes represent only one facet of many in perinatal experiences and outcomes. An alternate approach based on “asset-framing”—as opposed to “deficit framing”^3^—may reveal opportunities for novel interventions and strategies to promote optimal maternal health.

Studies on optimal birth outcomes remain sparse. While there is not yet a standardized definition of optimal birth, some studies have designed methods for measuring optimal births within specific populations. Although research on this topic varies in study design and population, themes center on the need for delivery of competent and respectful maternity care. For instance a measure of “uncomplicated pregnancies” (normotensive pregnancy, delivered at > 37 weeks, resulting in a liveborn baby who was not small for gestational age, and did not have any other significant pregnancy complications), a New Zealand study found that the majority of their study sample (61.6%) had an uncomplicated pregnancy^4^. An Iranian study defined a positive birth experience as an uncomplicated vaginal delivery with cephalic presentation and an overall healthy infant and found that survey respondents frequently mentioned themes of control, empowerment, coping, support, self-esteem, and self-efficacy to describe their birth experiences^5^. Similarly, a study of new mothers in Sweden used self-administered surveys and found that positive birth experiences depended on perceived self-efficacy and self-confidence, as well as the presence of personal advocates, such as close family members or midwives^6^.

In the experiences preceding, during, and following pregnancy, racism can lead to inequities in outcomes and experiences. The Centers for Disease Control and Prevention (CDC) estimated that during the 2007–2016 period, non-Hispanic Black and American Indian/Alaska Native (AIAN) individuals had significantly more pregnancy-related deaths per 100,000 births when compared to non-Hispanic White and Asian/Pacific Islander individuals^7^. Another CDC report estimated that in 2018, non-Hispanic Black infants had the highest mortality rate (10.75 per 100,000), followed by non-Hispanic Native Hawaiian and other Pacific Islanders (NHOPI) (9.39), non-Hispanic AIAN (8.15), Hispanic (4.86), non-Hispanic White (4.63), and non-Hispanic Asian (3.63) individuals^8^. Conceptually, racial equity involves theoretical differences in race, not just quantitative differences^9^. Maternal health researchers thusly argue for the need to achieve birth equity, defined as “the assurance of the conditions of optimal births for all people with a willingness to address racial and social inequities in a sustained effort”^10^.

To date, no population-level studies have focused on optimal reproductive health outcomes in the US, and none address the degree to which optimal reproductive health is (or is not) equitably experienced according to race and/or ethnicity. The purpose of this exploratory analysis was to measure the prevalence of optimal births in US counties from 2018 to 2019. We also sought to explore the spatial distribution of optimal births, in concordance with other public health studies who use area specific analyses of aggregated data to identify shared contextual factors among neighboring regions^11^. We further sought to estimate optimal birth prevalence by racial and ethnic identity and operationalize a county-level measure of birth equity.

## Methods

### Data sources and study population

This is a retrospective, ecologic and spatial analysis of county-level optimal birth rates. Data used in this report are from the National Center for Health Statistics (NCHS) restricted use natality files, 2018–2019. Raw data files were accessed following the granting of administrative permissions from the NCHS. These data include birth records for every birth occurring in the US, with Federal Information Processing System (FIPS) codes for identification of maternal county of residence in the 2019 US Census Bureau county-level shapefile. As this dataset contains only deidentified, secondary data, the study was deemed exempt by the Tulane University Institutional Review Board. All analyses were performed in accordance with the Declaration of Helsinki.

### Optimal birth

Optimal births were those that met the following criteria: live births without complications (gestational hypertension, eclampsia, or gestational diabetes), vaginal delivery at a gestational age of 37 weeks or later, and infant birth weight of > 2500 grams with no congenital anomalies (anencephaly, meningomyelocele/spina bifida, cyanotic congenital heart disease, congenital diaphragmatic hernia, omphalocele, gastroschisis, limb reduction defect, cleft palate/lip, down syndrome, suspected chromosomal disorder, hypospadias), emergency medical intervention beyond standard care (assisted ventilation, admission to NICU, surfactant use, antibiotic use, or seizures), and 5-min APGAR score ≥ 7^12^. The counts of birth records meeting these criteria in 2018 and 2019 were aggregated by county and divided by the sum of births occurring in the county over the same time period in order to estimate a raw rate of optimal births among live births. Additionally, a study by Jeon et. al found that it was best to racially stratify optimal birth weights and terms to account for differences in race^13^ and by subgroups based on maternal race/ethnicity (non-Hispanic White, non-Hispanic Black, and Hispanic). For this reason, we calculated both overall and race-specific rates of optimal births by county.

**Figure 1:**
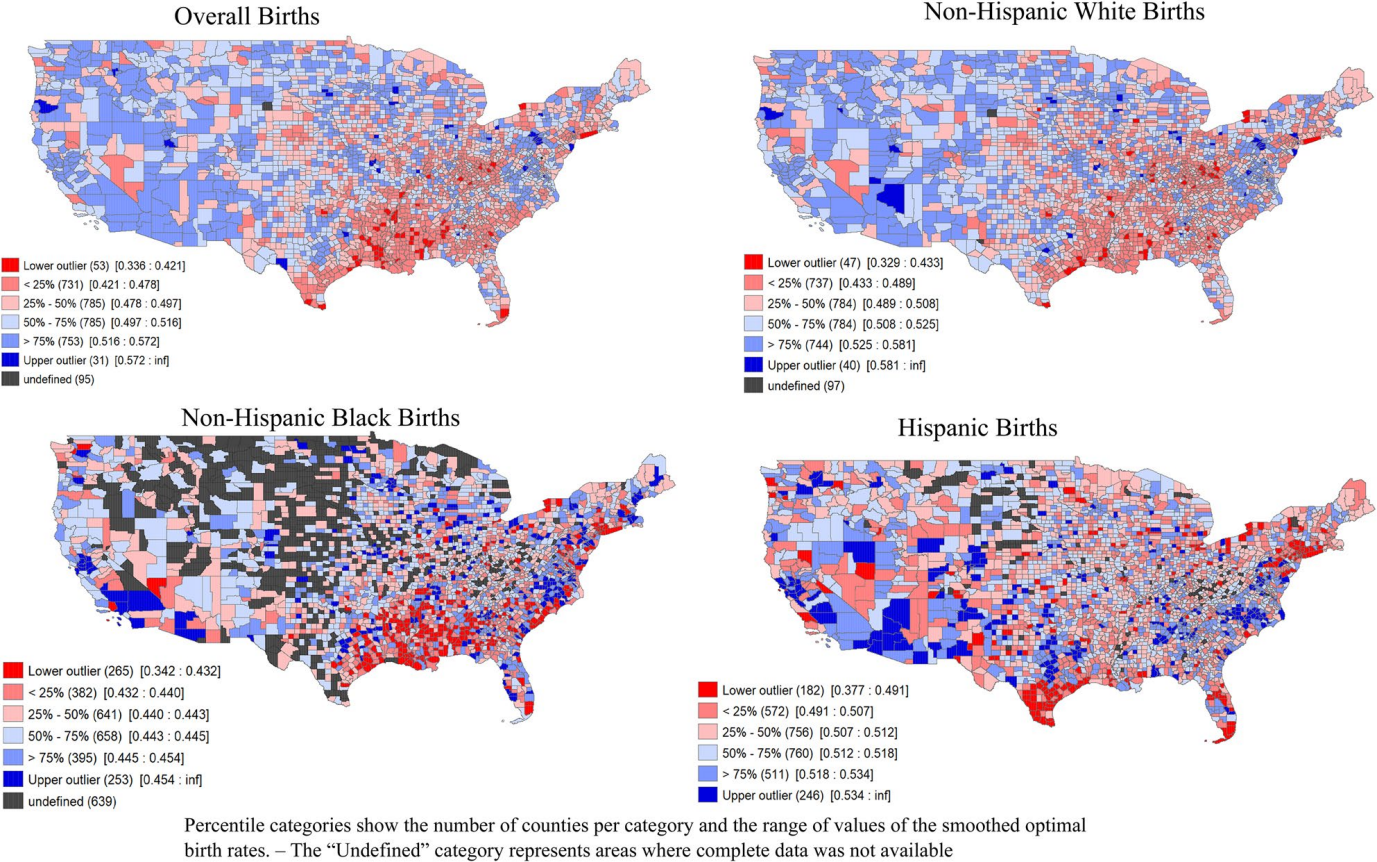
Overall and race-specific county-level empirical bayes smoothed rates of optimal births, 2018–2019. Map generated in Geoda version 1.18.0.10 (https://spatial.uchicago.edu/geoda).

Given the possibility of rate instability in sparsely populated counties, we calculated Empirical Bayes smoothed (EBS) rates of optimal birth for all live births and by maternal race/ethnicity by applying the smoothing tool in GeoDa version 1.18.0.10 (https://spatial.uchicago.edu/geoda). The empirical Bayes smoothing approach computes a weighted average between the raw estimate for each county and the national average, with weights proportional to the underlying population at risk^14,15^. As a result, spurious outliers (counties with small counts of births and large rate variance instability) are adjusted to a greater degree than counties with larger numbers of births and more stable rates. This method improves the precision of calculated rates by accounting for the high amount of population variance among counties in the United States by allowing geographic areas with smaller populations to “borrow strength” from those with larger populations in order to improve estimate precision^16^. In addition to the calculation of the smoothed rates, GeoDa was also used to generate corresponding maps of county level smoothed rates (Fig. 1, Figure S7).

### Birth equity

We defined counties achieving greater racial birth equity as those where the total population EBS optimal birth rate was higher than the national 75th percentile and the absolute difference between maternal racial/ethnic categories (non-Hispanic Black vs. non-Hispanic White and Hispanic vs. non-Hispanic White) was smaller than the national 25th percentile difference. While using White as a reference group is not always appropriate—due to the group’s increasing heterogeneity and the possibility of further entrenching existing racial inequities^17^—this analysis uses it only in a descriptive manner to estimate the disparity—that is the numeric difference between two populations—of optimal births between Whites, who comprise the majority of the US population, and Blacks and Hispanics, two populations within whom heightened risks exist for adverse outcomes. In order to visualize this measure, maps were created in ArcMap 10.7 showing the estimated health-equity status of each county.

### Statistical analysis

**Figure 2:**
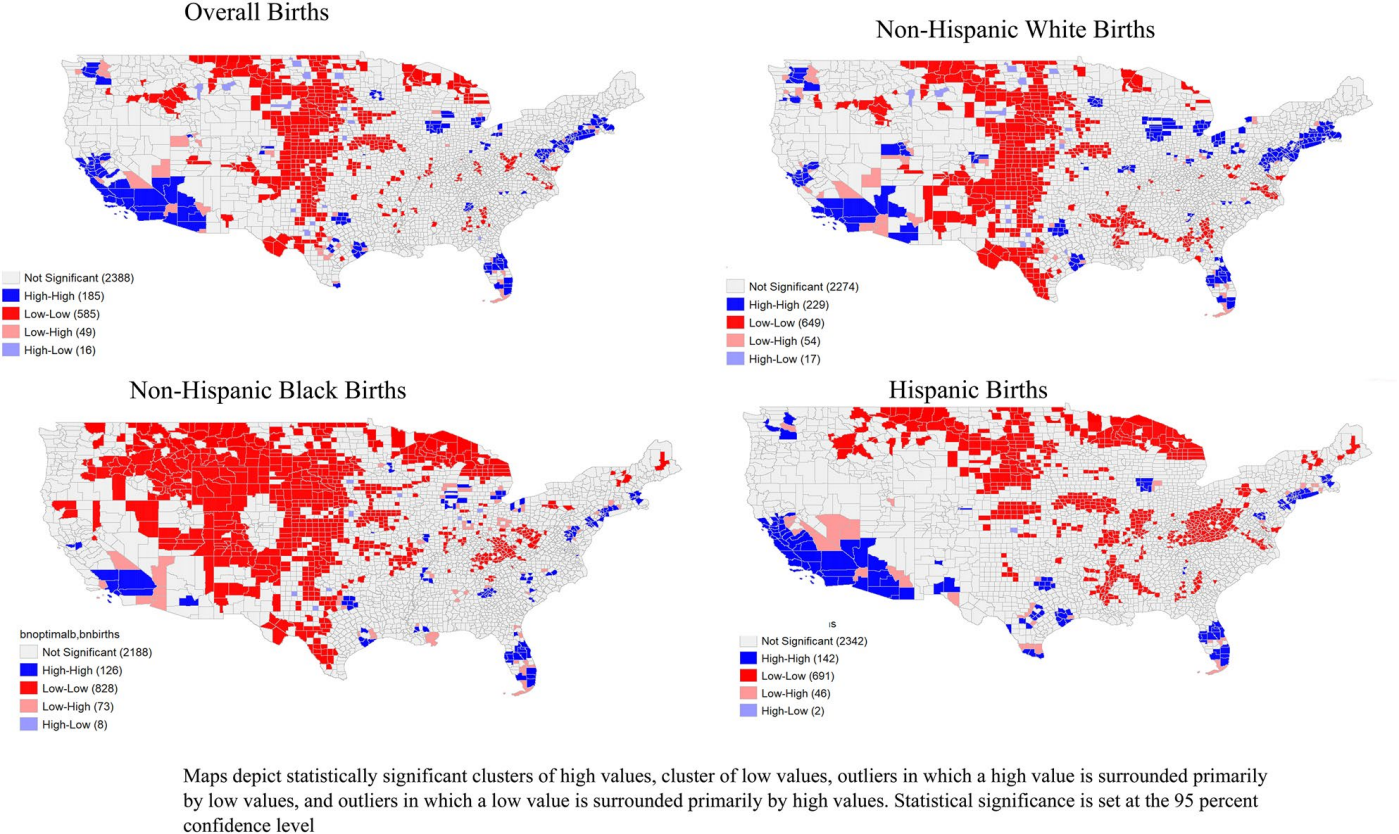
Overall and race-specific county-level local-indicators of spatial autocorrelation (LISA) cluster significance using raw rates of optimal births. Map generated in Geoda version 1.18.0.10 (https://spatial.uchicago.edu/geoda).

Frequencies were calculated for categorical maternal characteristics—both overall and by optimal birth status. For maternal characteristics that were continuous variables, the mean, median, standard deviation, 25th percentile, and 75th percentile values were calculated. Spatial analyses were conducted to map all EBS rates by county, as well as the indicator for birth equity. We ran Global Moran’s I tests to examine clustering of birth equity across counties and Local Indicators of Spatial Autocorrelation (LISA) for local clustering of the raw rates of optimal births to identify clusters of counties with similar characteristics (Fig. 2). We used first-order queen contiguity spatial weights and 999 permutations for statistical inference in Local Moran’s I analyses at the 95% confidence level, all of which were conducted in GeoDa^18^.

The issue of multiple comparisons is a common methodological concern associated with local spatial autocorrelation statistics. In addition to Fig. 2, we include maps for areas of statistically significant clustering based on a Simes adjusted p value^19^ in supplemental Figure S7 to further reveal potentially salient locations that warrant more extensive sensitivity analyses in future work.

## Results

There were 7,520,961 live births occurring from 2018 to 2019, 49.80% of which were classified as optimal births, according to the definition described in the study methods. A full descriptive depiction of both categorical and continuous variables is shown in Table 1.

**Table 1:** Optimal birth outcome and maternal birth record characteristic descriptive statistics (2018–2019).

Characteristic	N (%)	N (%)	N (%)		
	Total births 7,520,961 (100)	Non-optimal births 3,775,785 (50.20)	Optimal births 3,745,176 (49.80)		
**Maternal race**					
Black	1,091,721 (14.65)	608,229 (16.27)	483,492 (13.03)		
Hispanic	1,770,027 (23.75)	864,030 (23.11)	905,997 (24.41)		
White	3,866,300 (51.89)	1,886,572 (50.45)	1,979,728 (53.34)		
Other	723,289 (9.71)	380,645 (10.18)	342,644 (9.23)		
**Preterm birth**					
Yes	760,963(10.12)	760,963 (20.18)	0 (0)		
No	6,755,093 (89.88)	3,009,917 (79.82)	3,745,176 (100.00)		
**Low birth weight**					
Yes	765,868 (10.19)	629,775 (16.68)	0 (0)		
No	6,755,093 (89.82)	3,146,010 (83.32)	3,745,176 (100.00)		
**Gestational diabetes**					
Yes	510,426 (6.79)	510,426 (13.54)	0 (0)		
No	7,004,375 (93.21)	3,259,199 (86.46)	3,745,176 (100.00)		
**Gestational hypertension**					
Yes	561,921 (7.48)	561,921 (14.91)	0 (0)		
No	6,952,880 (92.52)	3,207,704 (85.09)	3,745,176 (100.00)		
**Need for emergency intervention**					
Yes	845,093 (11.25)	845,093 (22.42)	0 (0)		
No	6,669,081 (88.75)	2,923,905 (77.58)	3,745,176 (100.00)		
**Congenital anomalies**					
Yes	25,922 (0.35)	25,922 (0.69)	0 (0)		
No	7,483,638 (99.65)	3,738,462 (99.31)	3,745,176 (100.00)		
**APGAR score greater than or equal to 7**					
Yes	181,652 (2.42)	3,594,133 (95.19)	0 (0)		
No	7,339,309 (97.58)	181,652 (4.81)	3,745,176 (100.00)		
**Fetal presentation**					
Spontaneous	4,899,565 (65.18)	1,154,389 (30.60)	3,745,176 (100)		
Forceps	38,284 (0.51)	38,284 (1.01)	0 (0)		
Vacuum	190,540 (2.53)	190,540 (5.05)	0 (0)		
Cesarean	2,388,773 (31.78)	2,388,773 (63.33)	0 (0)		

	Mean	Median	Standard deviation	25th percentile	75th percentile
**Race stratified county-level percent optimal**					
Overall	49.73	49.64	6.87	45.76	53.65
Black	45.21	44.55	24.52	35.71	51.32
White	50.44	50.25	7.87	46.33	54.55
Hispanic	50.99	50.55	21.02	43.18	59.26
Other	47.15	48.25	24.24	36.00	57.74
**Race specific equity measure (absolute difference in optimal births)**					
White to black	16.06	8.69	17.04	4.16	20.65
White to Hispanic	11.23	6.36	13.09	2.57	14.41

The majority of live births during the study period resulted in a normal term birth, (79.72%), normal birthweight (89.82%), no gestational diabetes (93.21%), no gestational hypertension (92.52%), no abnormal conditions (88.90%), no need for emergency interventions (88.75%), a vaginal delivery (65.18%), and 97.58% had a five-minute APGAR score greater than or equal to 7. Race-stratified univariate results demonstrated some differences in the prevalence of optimal births among non-Hispanic Black (45.21%), compared to non-Hispanic White (50.44%) or Hispanic (50.99%) births. The White and Hispanic maternal race groups show a higher proportion of optimal births within their respective racial categories when compared to the lower proportion shown among the Black and Other categories.

LISA estimates identified statistically significant spatial clusters including counties with high rates of optimal birth that were surrounded by neighboring counties with high rates of optimal birth (high-high clusters), counties with low rates of optimal birth surrounded by neighboring counties with low rates of optimal birth (low-low clusters) and outliers on both ends (counties with a high rate of optimal birth surrounded by neighboring counties with low rates of optimal birth and counties with a low rate of optimal birth surrounded by neighboring counties with high rates of optimal birth).

Figure 1 displays the distribution of EBS optimal birth rates across US counties. Many of the counties concentrated in the southeastern US had low prevalence of optimal births. Mapped county level EBS rates among White individuals most similarly mirrored the map showing EBS rates for the overall population, as is to be expected as the largest racial group. Hispanic EBS rates appeared to be particularly poor in the counties along the Texas-Mexico border. In the analysis of spatial autocorrelation with LISA, we estimated positive, significant clustering of optimal births in the overall population by county indicating the presence of nonrandom spatial distribution (Moran’s I: 0.471). Figure 2 identifies where significant clustering occurred using raw rates of optimal births. Among Black births, the southeastern US counties have a greater number of low-low clusters for optimal births, stretching mostly along the gulf coast states. The map showing the spatial distribution of for those whose race was classified as “Other” is included in the supplemental material (Figures S5) in addition to the cluster maps (Figure S6). Supplemental Figure S7 displays significant clustering with Simes adjusted p values.

**Figure 3:**
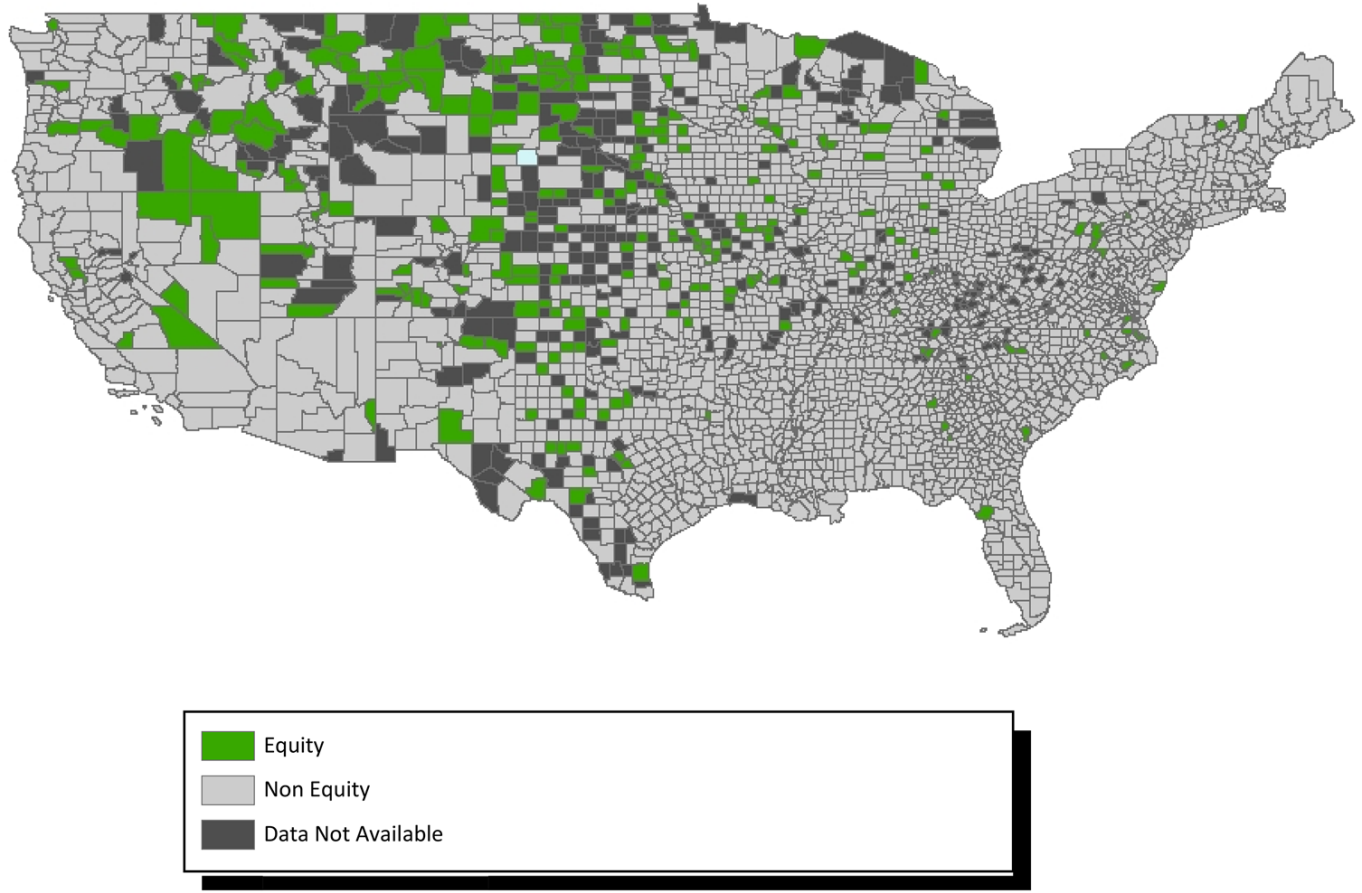
County level racial equity in optimal births—white to black. Map generated in ArcMap 10.7 (https://www.esri.com).

**Figure 4:**
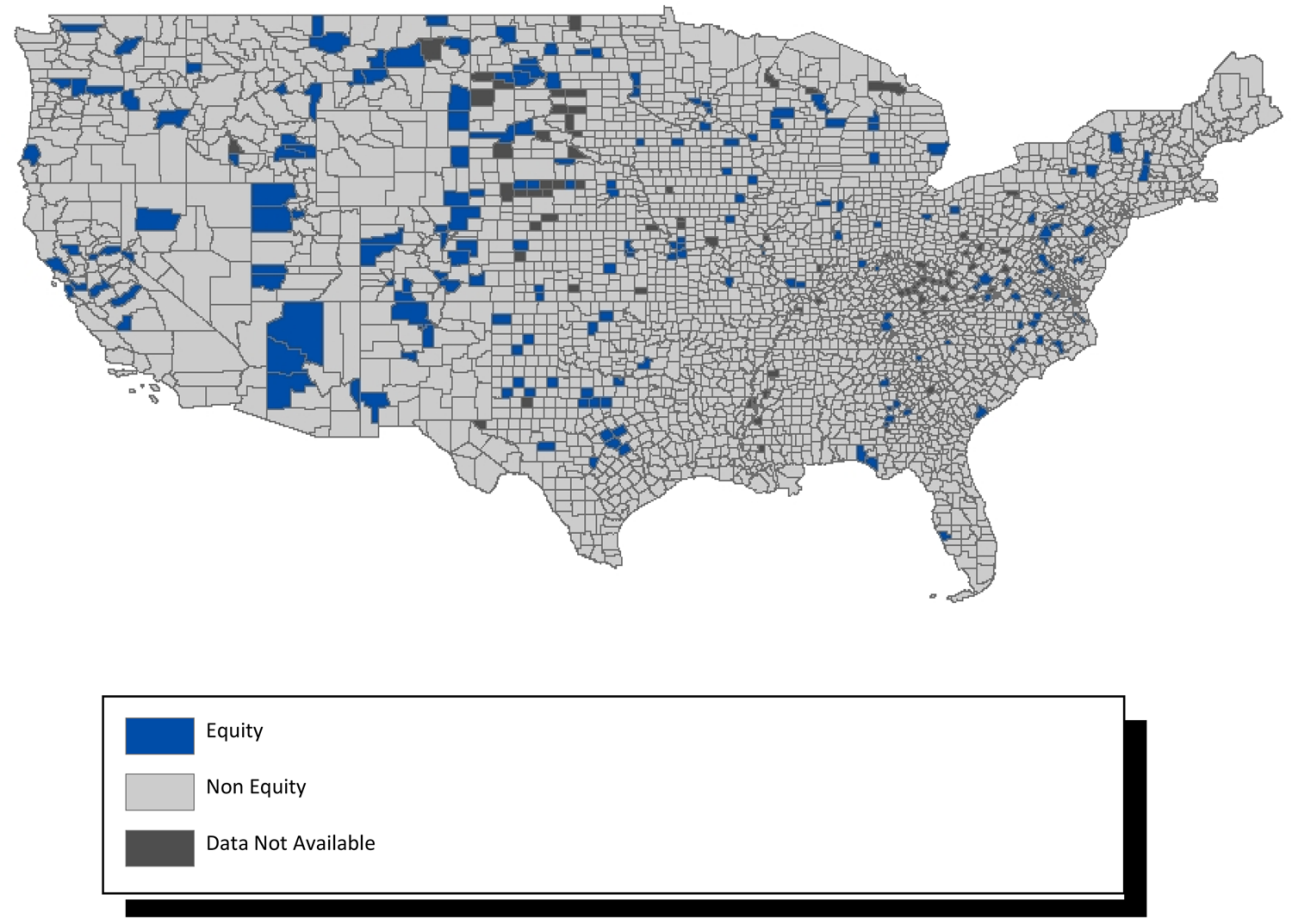
County level racial equity in optimal births—white to hispanic. Map generated in ArcMap 10.7 (https://www.esri.com).

Of the 3140 census-designated counties within the US, only 282 (8.98%) appeared to be advancing equity in optimal births between White and Black populations (Fig. 3), and 205 (6.53%) were estimated to be advancing equity in optimal births between White and Hispanic populations (Fig. 4). A list of these counties appears in supplemental Table S2. Figure 3 shows that counties advancing between white-Black births to be particularly concentrated in the Midwestern states.

## Discussion

In order to contribute a new perspective to the discussion on reproductive health in the US, this study developed an optimal birth outcome measure and studied its spatial distribution to create a novel measure of birth equity. The purpose of this exploratory analysis was to define an optimal birth outcome measure, identify its spatial distribution, and create a novel measure of birth equity in order to contribute a new perspective to the discussion on reproductive health in the US. This study is the first to use a single measure to quantify the prevalence of optimal births within the United States. In a univariate analysis of the overall population during the 2018–2019 period, we found that slightly less than half of all births could be classified as having been optimal according to the selected criteria. The prevalence estimates of optimal births stratified by race showed that no racial/ethnic category maintained a majority of optimal births.

The results of this analysis showed county-level differences in the racial equity of optimal births when comparing the White population to the Hispanic population and separately to the Black population. In measuring adverse birth outcomes there is often a wide margin in the difference between race-specific results. However, this analysis showed that the racial inequity in the prevalence of optimal births—while nonzero—had much smaller margins in comparison. By conducting this analysis at this level of geography, we can use the principle of homogeneity to infer that findings within a county might be reflective of regional similarities^20^. A World Health Organization report distinguished social inequities in health from mere variations or differences in health by noting the presence of factors that are systematic, socially produced, and unfair^21^. Unlike an immutable characteristic like individual race^22^, racial systemic inequality is a factor which can be addressed through policy intervention. Similarly, by identifying counties with birth equity, approaches to address racial/ethnic inequity in pregnancy and birth outcomes can be developed.

The study’s strengths include the possible reduction of the influence of outliers due to the large sample size and the utilization of EBSR, and the geographic dimension added by the analysis of spatial autocorrelation. However, this study also has several weaknesses. Vital records are the only national source of data on birth outcomes, including geographic identifiers for maternal residence. Still, our reliance on vital records data is the most profound limitation of this analysis. We defined optimal birth using all relevant variables available, but vital records fail to capture many aspects of pregnancy and childbirth that define optimal experiences. These include and are not limited to indicators of maternal mental health and well-being, the quality and respectfulness of maternity care, the absence of discriminatory treatment in medical and other institutional and social settings, and the degree to which people have the power to make unconstrained choices about whether or not to carry a pregnancy to birth and the circumstances (where and with whom) in which the birth occurs. Additionally, some of the birth records were missing data on the variables used in the optimal births measure. Because this is a county-level analysis, small counts of births prohibited our stratifying by maternal race/ethnicity beyond the three groups we included, or further stratifying within these non-monolithic categorizations. This statistical problem does not undermine the need for research and interventions to improve optimal birth and advance birth equity among other racial and ethnic identities. The concentration of counties achieving equity in the Midwest could be due to low Black and Hispanic population counts compared to White which may have led to overestimations. Lastly, because this is an ecologic and spatial analysis of the distribution of optimal birth rates, we are careful not to draw any conclusions about individual-level risk.

Increased focus on optimal birth outcomes could reveal opportunities for improvement of care procedures in the perinatal period. A systematic review identified factors related to empowerment during the perinatal period, namely perceived control of decision-making and resources, as well as belief in one’s ability^23^. In order to guarantee optimal outcomes, all aspects of the birth process should be addressed. Future analyses should continue to explore and monitor optimal birth and its correlates in order to broaden our understanding of reproductive health in the US. While this analysis is descriptive in nature, multivariate models may reveal associations between optimal births and sociocontextual, environmental, and/or policy factors that may be manipulated through intervention for the purpose of advancing racial health equity. Additionally, longitudinal analyses could show how optimal births change over time, especially as the healthcare landscape continues to evolve with new economic and political policies motivated by a growing acknowledgement of the role that medical and structural racism play in maintaining racial health inequities.

## Conclusion

In the effort improve maternal health, we should focus not only on the absence of negative outcomes, but also the occurrence of positive outcomes, a paradigm shift that may prove insightful and effective^24^. Our analytic results suggest that optimal births can be measured and that spatial patterns exist at the county level for this outcome.

## Supplementary Information

Supplementary Information.

## Data Availability

The data that support the findings of this study are available from the National Center for Health Statistics, but restrictions apply to the availability of these data, which were used under license for the current study, and so are not publicly available. Aggregated data are however available from the corresponding author upon reasonable request and with permission of the National Center for Health Statistics.
