# Supplementary material for: US county-level prevalence and spatial distribution of optimal birth outcomes 2018–2019
Source: Sci Rep. 2022 Oct 3;12:16535. doi: 10.1038/s41598-022-20517-9 (PMC9529881; doi:10.1038/s41598-022-20517-9)
Supplement: Supplementary file 1 — Supplementary Information. [file 41598_2022_20517_MOESM1_ESM.docx]

Table S2: Counties that attained equity in Optimal births between White/Black population

| **County Name** | **State** |
| --- | --- |
| BRISTOL BAY BOROUGH | AK |
| DENALI | AK |
| DILLINGHAM | AK |
| FAIRBANKS NORTH STAR | AK |
| HAINES | AK |
| HOONAH-ANGOON CENSUS AREA | AK |
| LAKE AND PENINSULA | AK |
| PR.OF WALES-HYDER CNS AREA | AK |
| GREENLEE | AZ |
| COLUSA | CA |
| INYO | CA |
| KINGS | CA |
| YOLO | CA |
| BACA | CO |
| CHEYENNE | CO |
| CUSTER | CO |
| DELTA | CO |
| HINSDALE | CO |
| JACKSON | CO |
| KIOWA | CO |
| LINCOLN | CO |
| MINERAL | CO |
| MORGAN | CO |
| OURAY | CO |
| SAN JUAN | CO |
| SAN MIGUEL | CO |
| SEDGWICK | CO |
| TELLER | CO |
| WELD | CO |
| LEVY | FL |
| CARROLL | GA |
| HABERSHAM | GA |
| SCHLEY | GA |
| TALBOT | GA |
| BOUNDARY | ID |
| CUSTER | ID |
| GOODING | ID |
| JEROME | ID |
| LEMHI | ID |
| LEWIS | ID |
| NEZ PERCE | ID |
| ONEIDA | ID |
| OWYHEE | ID |
| POWER | ID |
| VALLEY | ID |
| WASHINGTON | ID |
| CLAY | IL |
| JASPER | IL |
| KANKAKEE | IL |
| MERCER | IL |
| SHELBY | IL |
| BOONE | IN |
| JAY | IN |
| NOBLE | IN |
| SHELBY | IN |
| SWITZERLAND | IN |
| TIPPECANOE | IN |
| WASHINGTON | IN |
| GRUNDY | IA |
| IOWA | IA |
| LUCAS | IA |
| MILLS | IA |
| VAN BUREN | IA |
| WAYNE | IA |
| ANDERSON | KS |
| CHASE | KS |
| CHAUTAUQUA | KS |
| COMANCHE | KS |
| DONIPHAN | KS |
| ELLSWORTH | KS |
| GEARY | KS |
| HASKELL | KS |
| JEWELL | KS |
| LINN | KS |
| LOGAN | KS |
| MEADE | KS |
| STANTON | KS |
| STEVENS | KS |
| WICHITA | KS |
| WILSON | KS |
| WYANDOTTE | KS |
| BUTLER | KY |
| WASHINGTON | MD |
| WORCESTER | MD |
| BARRY | MI |
| JACKSON | MI |
| LUCE | MI |
| MECOSTA | MI |
| OTSEGO | MI |
| ANOKA | MN |
| CLEARWATER | MN |
| COOK | MN |
| FILLMORE | MN |
| LAC QUI PARLE | MN |
| LYON | MN |
| NOBLES | MN |
| POLK | MN |
| WILKIN | MN |
| ANDREW | MO |
| ATCHISON | MO |
| BUCHANAN | MO |
| CEDAR | MO |
| CLAY | MO |
| CLINTON | MO |
| DAVIESS | MO |
| HOLT | MO |
| KNOX | MO |
| MONITEAU | MO |
| MORGAN | MO |
| OREGON | MO |
| OSAGE | MO |
| PUTNAM | MO |
| ST. CLAIR | MO |
| SCHUYLER | MO |
| SCOTLAND | MO |
| CARTER | MT |
| CASCADE | MT |
| CHOUTEAU | MT |
| DANIELS | MT |
| GARFIELD | MT |
| GOLDEN VALLEY | MT |
| GRANITE | MT |
| JEFFERSON | MT |
| JUDITH BASIN | MT |
| LIBERTY | MT |
| LINCOLN | MT |
| MINERAL | MT |
| MUSSELSHELL | MT |
| PETROLEUM | MT |
| POWDER RIVER | MT |
| ROSEBUD | MT |
| SANDERS | MT |
| STILLWATER | MT |
| TREASURE | MT |
| VALLEY | MT |
| WHEATLAND | MT |
| WIBAUX | MT |
| YELLOWSTONE | MT |
| ANTELOPE | NE |
| BANNER | NE |
| CEDAR | NE |
| CUMING | NE |
| DEUEL | NE |
| DIXON | NE |
| DODGE | NE |
| GRANT | NE |
| HAYES | NE |
| HOOKER | NE |
| JOHNSON | NE |
| KEYA PAHA | NE |
| LOUP | NE |
| PAWNEE | NE |
| SIOUX | NE |
| THAYER | NE |
| VALLEY | NE |
| WHEELER | NE |
| ELKO | NV |
| ESMERALDA | NV |
| EUREKA | NV |
| HUMBOLDT | NV |
| HARDING | NM |
| LOS ALAMOS | NM |
| MORA | NM |
| OTERO | NM |
| BURKE | NC |
| CAMDEN | NC |
| CATAWBA | NC |
| GATES | NC |
| GREENE | NC |
| PAMLICO | NC |
| PASQUOTANK | NC |
| VANCE | NC |
| BILLINGS | ND |
| BOTTINEAU | ND |
| BOWMAN | ND |
| BURKE | ND |
| BURLEIGH | ND |
| DUNN | ND |
| EDDY | ND |
| EMMONS | ND |
| FOSTER | ND |
| GOLDEN VALLEY | ND |
| GRANT | ND |
| GRIGGS | ND |
| HETTINGER | ND |
| LA MOURE | ND |
| LOGAN | ND |
| MCHENRY | ND |
| MERCER | ND |
| MORTON | ND |
| NELSON | ND |
| OLIVER | ND |
| PEMBINA | ND |
| RENVILLE | ND |
| SHERIDAN | ND |
| SIOUX | ND |
| STEELE | ND |
| WARD | ND |
| ALFALFA | OK |
| BEAVER | OK |
| DEWEY | OK |
| NOWATA | OK |
| CROOK | OR |
| GRANT | OR |
| JEFFERSON | OR |
| LINN | OR |
| MALHEUR | OR |
| CAMBRIA | PA |
| FULTON | PA |
| HUNTINGDON | PA |
| MONTOUR | PA |
| JASPER | SC |
| BON HOMME | SD |
| BROOKINGS | SD |
| BUTTE | SD |
| CLARK | SD |
| CUSTER | SD |
| DEUEL | SD |
| DEWEY | SD |
| HAAKON | SD |
| HANSON | SD |
| HUGHES | SD |
| HUTCHINSON | SD |
| KINGSBURY | SD |
| MOODY | SD |
| PERKINS | SD |
|  |  |
| BLEDSOE | TN |
| FENTRESS | TN |
| VAN BUREN | TN |
| ARCHER | TX |
| CARSON | TX |
| CLAY | TX |
| COKE | TX |
| EDWARDS | TX |
| GLASSCOCK | TX |
| HARTLEY | TX |
| HEMPHILL | TX |
| KENEDY | TX |
| KING | TX |
| LAMPASAS | TX |
| MENARD | TX |
| MILLS | TX |
| MORRIS | TX |
| MOTLEY | TX |
| RANDALL | TX |
| ROBERTS | TX |
| STERLING | TX |
| STONEWALL | TX |
| TERRELL | TX |
| TERRY | TX |
| THROCKMORTON | TX |
| BEAVER | UT |
| JUAB | UT |
| KANE | UT |
| MORGAN | UT |
| RICH | UT |
| ESSEX | VT |
| LAMOILLE | VT |
| ISLE OF WIGHT | VA |
|  |  |
| EMPORIA CITY | VA |
| MANASSAS PARK CITY | VA |
| RADFORD CITY | VA |
| COWLITZ | WA |
| GARFIELD | WA |
| SAN JUAN | WA |
| WAHKIAKUM | WA |
| ADAMS | WI |
| BROWN | WI |
| BUFFALO | WI |
| BURNETT | WI |
| KEWAUNEE | WI |
| LA CROSSE | WI |
| LAFAYETTE | WI |
| MARQUETTE | WI |
| OZAUKEE | WI |
| PEPIN | WI |
| PIERCE | WI |
| SAWYER | WI |
| CROOK | WY |
| UINTA | WY |

Figure S5: County-level empirical bayes smoothed rates of optimal births for “Other” race, 2018-2019


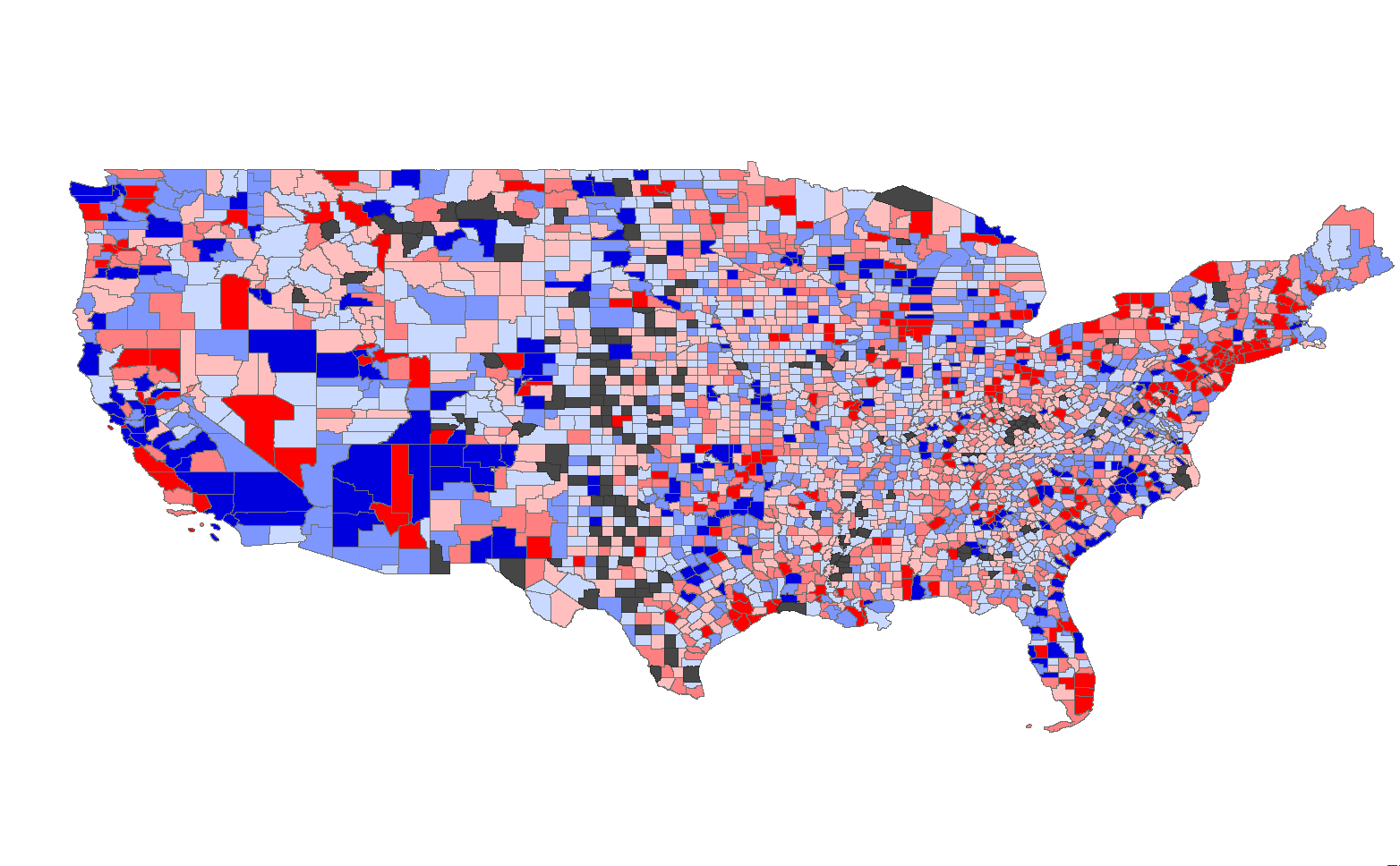

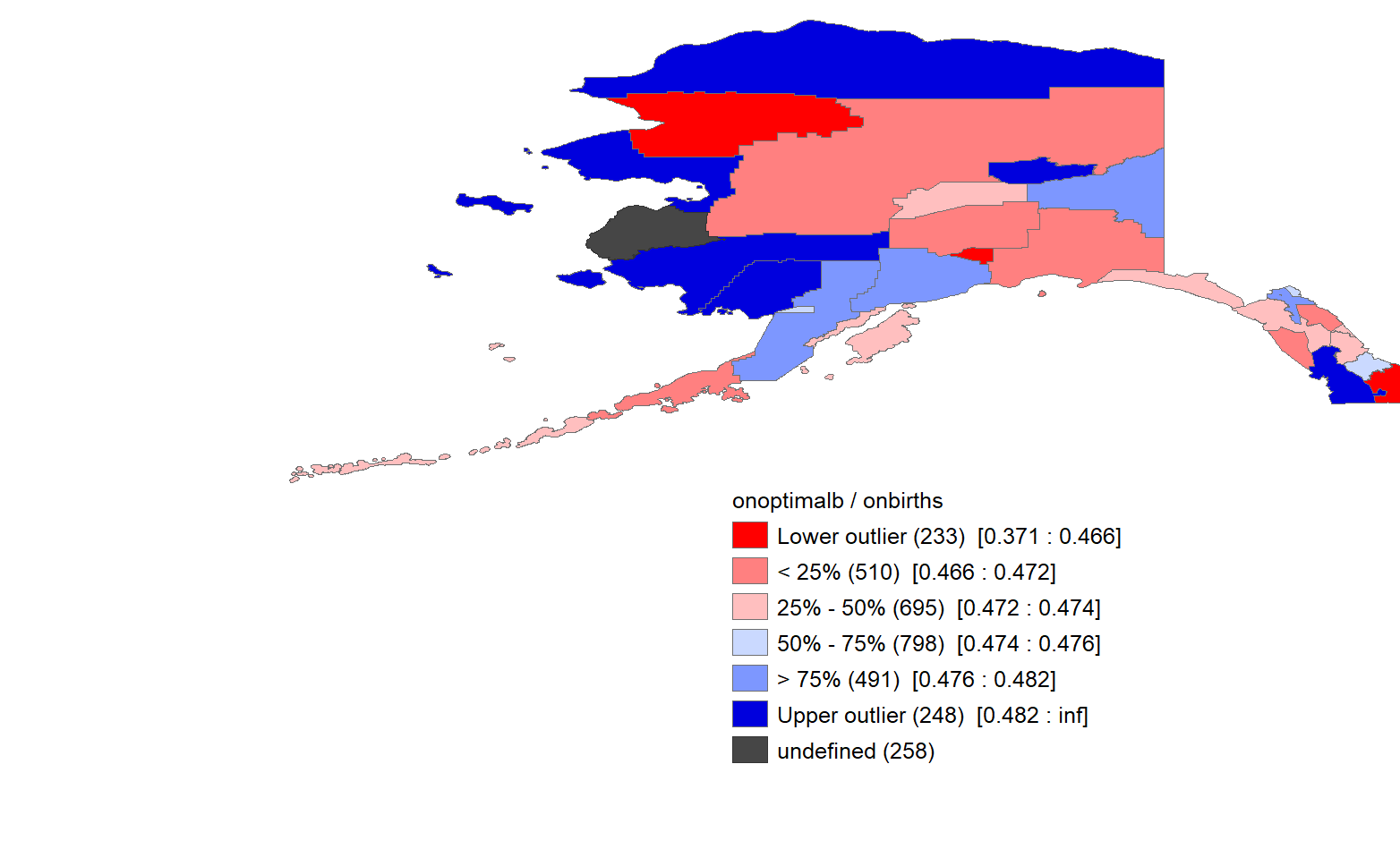


*Map generated in Geoda version 1.18.0.10 (*[*https://spatial.uchicago.edu/geoda*](https://spatial.uchicago.edu/geoda)*)*

Figure S6: County-level Local-Indicators of Spatial Autocorrelation (LISA) cluster significance for “Other” race, 2018-2019


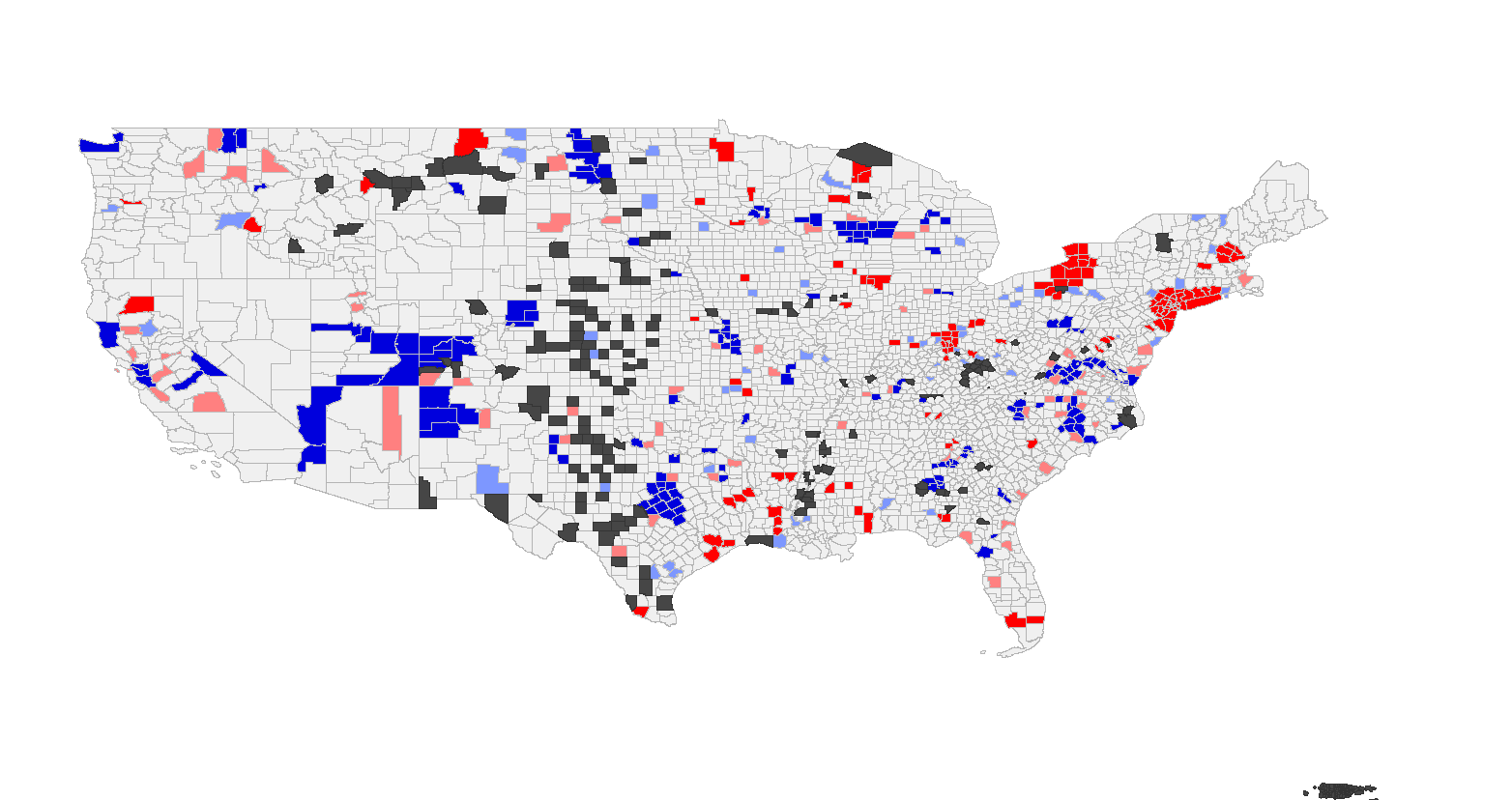

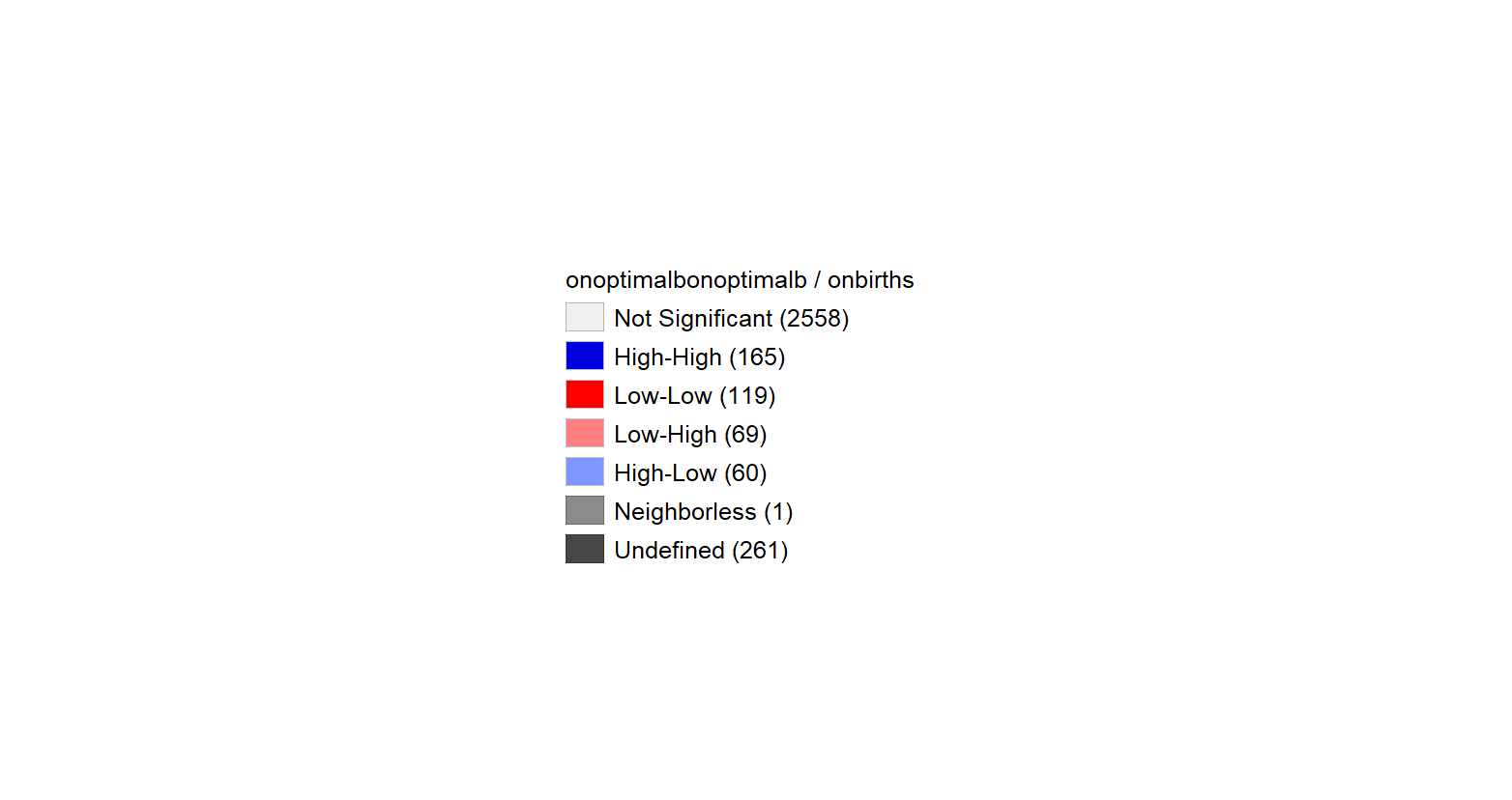


*Map generated in Geoda version 1.18.0.10 (*[*https://spatial.uchicago.edu/geoda*](https://spatial.uchicago.edu/geoda)*)*

Figure S7: Simes-adjusted overall and race-specific county-level Local-Indicators of Spatial Autocorrelation (LISA) cluster significance using raw rates of optimal births.

Non-Hispanic Black Births

Non-Hispanic White Births

Overall Births


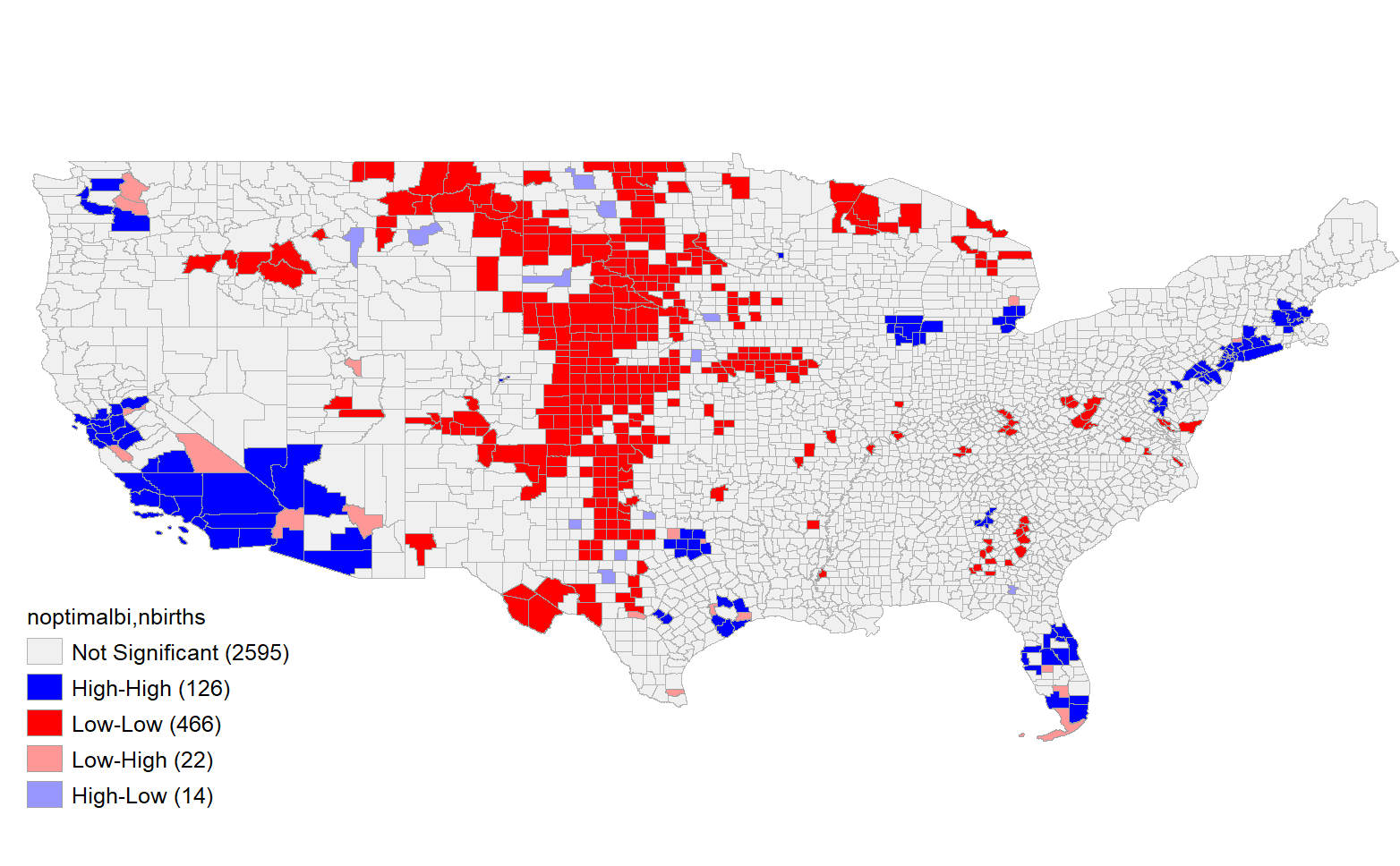

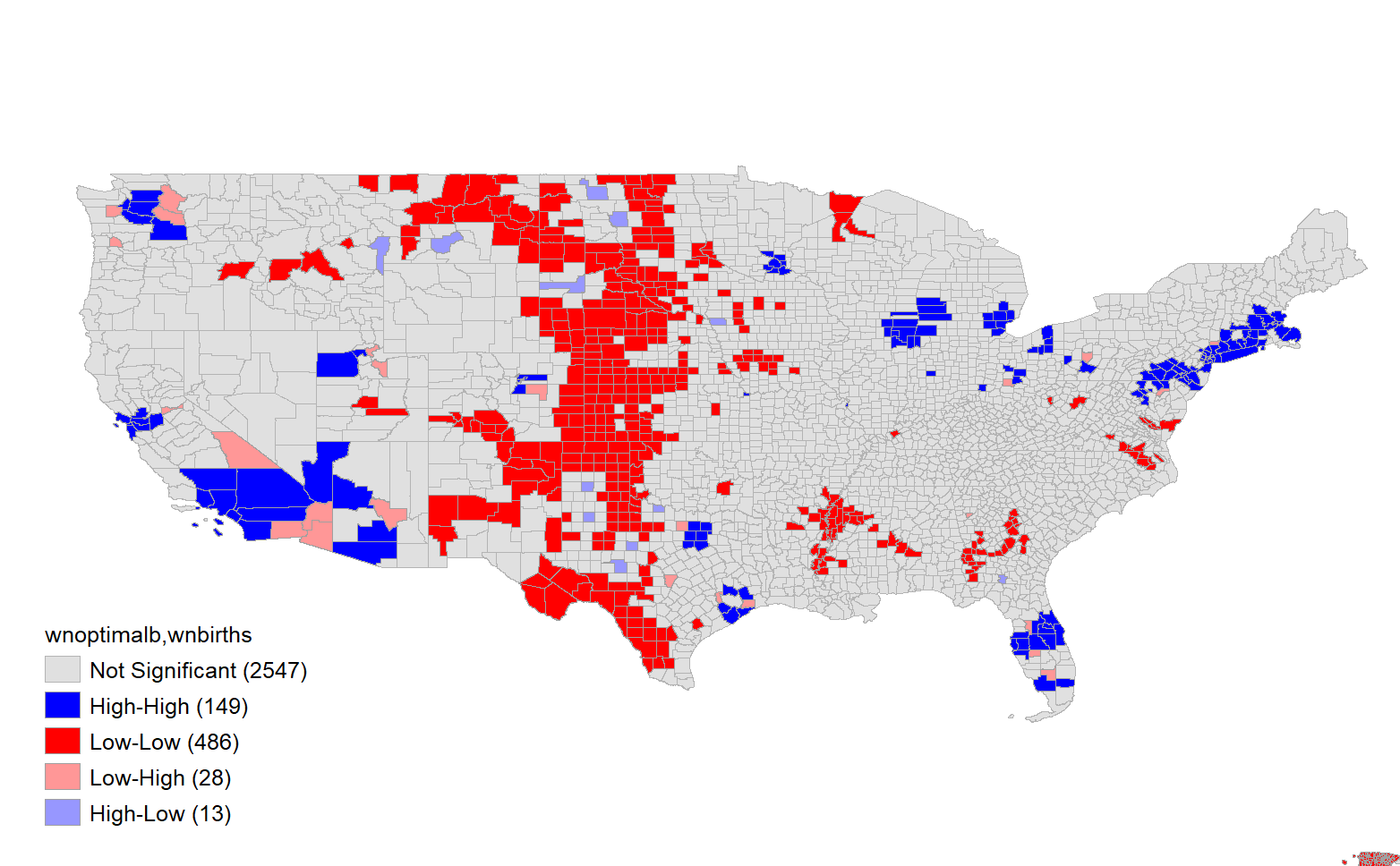

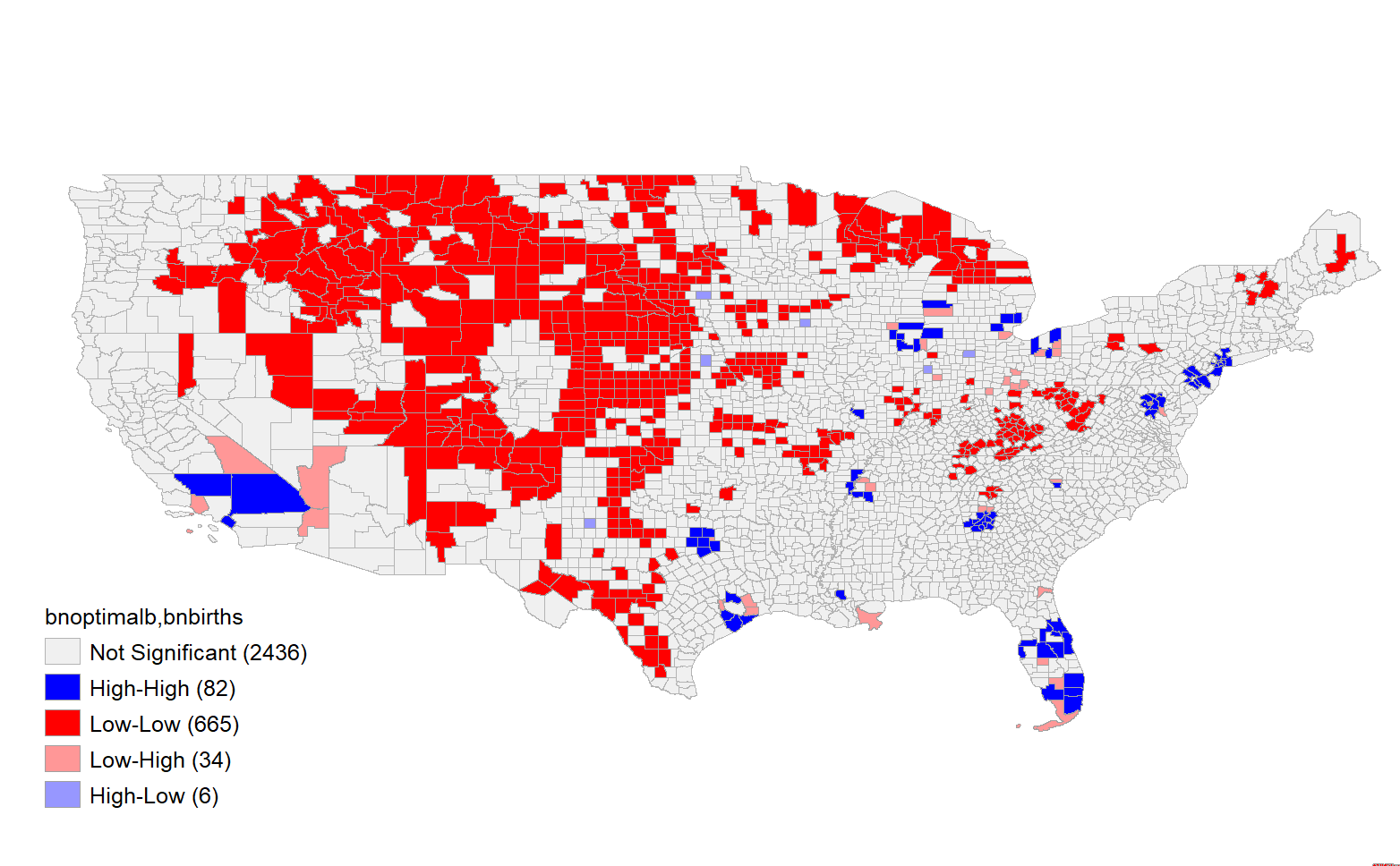

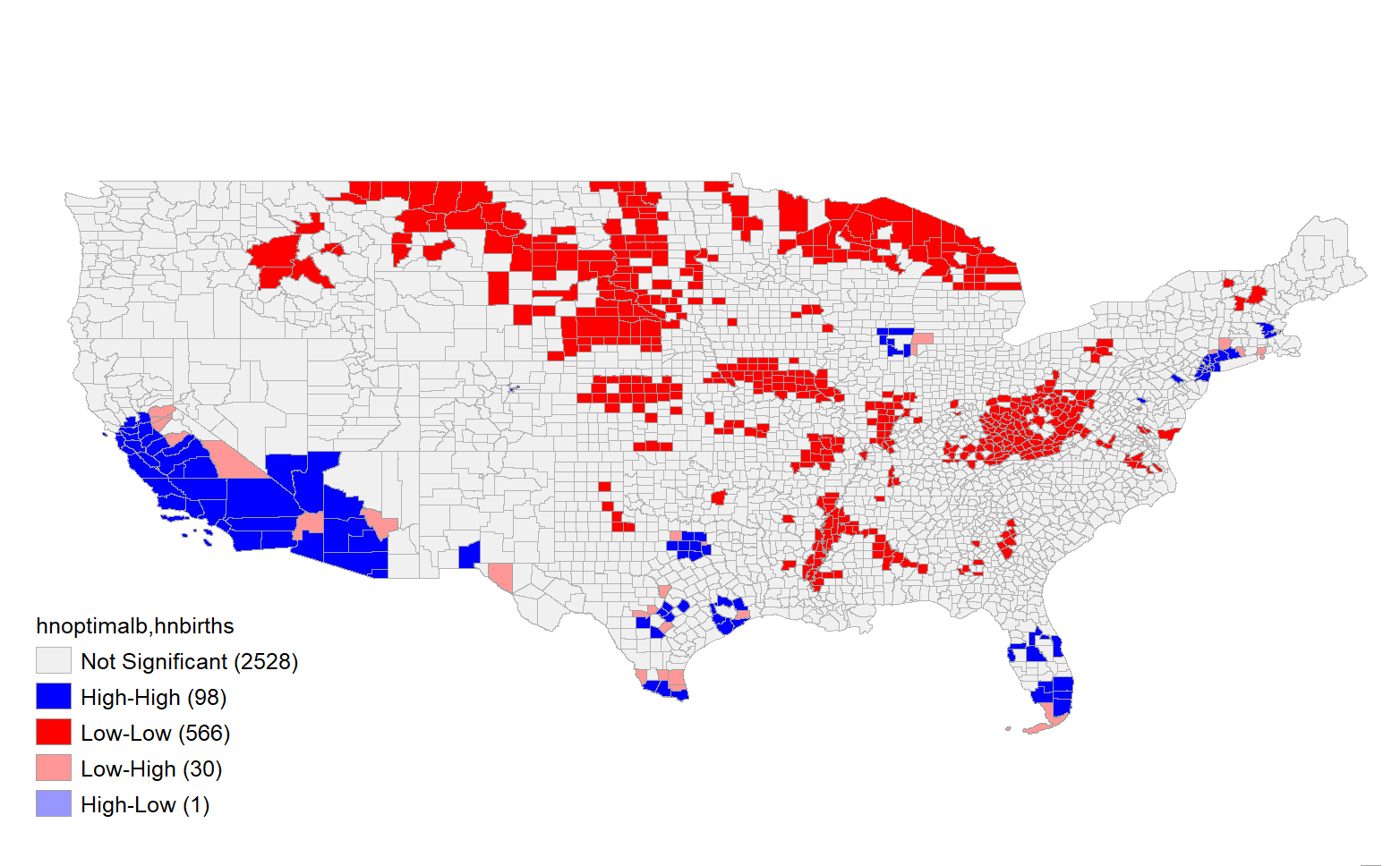


Hispanic Births

Maps depict statistically significant clusters of high values, cluster of low values, outliers in which a high value is surrounded primarily by low values, and outliers in which a low value is surrounded primarily by high values. Statistical significance is set at the 95 percent confidence level

*Map generated in Geoda version 1.18.0.10 (*[*https://spatial.uchicago.edu/geoda*](https://spatial.uchicago.edu/geoda)*)*
